# Transcriptomic data-mining approach for identifying potential pharmacogenetic candidates in antiepileptic drug response

**DOI:** 10.4103/0971-6866.80361

**Published:** 2011-05

**Authors:** Abhay Sharma

**Affiliations:** Institute of Genomics and Integrative Biology, Council of Scientific and Industrial Research, Delhi University Campus, Mall Road, Delhi - 110 007, India

Sir,

Antiepileptic drug (AED) therapy is known to be associated with significantly high rates of adverse reactions and ineffective seizure control in a considerable proportion of epileptic patients.[1] Currently in its infancy,[2,3] pharmacogenomics of AED response therefore constitutes a priority area in the field of personalized medicine. Although interest in the genetic association of adverse drug reactions and efficacy is gradually increasing, hypothesis-free genome-wide association study (GWAS) approaches in epilepsy have not become a reality yet.[2,3] Candidate-based association approaches will thus remain the mainstay in the area of epilepsy genetics and AED pharmacogenetics in the immediate future. Success of such approaches will however depend much on the criteria used for selecting the candidate genes. Identifying promising candidate genes will not only enhance the chances of success in candidate-based association studies but will also assist in the analysis of GWAS results, once available. Here, I examine whether transcriptomic data-mining could facilitate identification of potential pharmacogenetic candidate genes in AED therapy. I have selected sodium valproate (NaVP), a drug prescribed widely in epilepsy and mood disorders, for my analysis mainly due to the availability of multiple microarray gene expression results for this AED in the literature. I specifically examined whether the genes reported as differentially expressed after NaVP treatment show statistically significant enrichment of gene ontology biological processes related to central nervous system function. Further, I examined whether differentially expressed genes also over-represent the reported AED pharmacogenetic candidates.

Four reports on NaVP were identified as relevant in the context of brain or neuronal function – rat brain, 30-day treatment[4] (gene list, *pers comm*.); mouse brain, 7-day treatment;[5] cultured rat cortical neurons, 12-h treatment;[6] human neuroblastoma cells, 6- and 72-h treatments.[7] These diverse studies reported differentially expressed genes with insignificant overlaps. The genes were therefore pooled together for downstream analysis, total up- and down-regulated genes being 817 and 360, in that order. The Functional Annotation Tool in DAVID[8,9] was used to examine the over-represented processes in up- and down-regulated genes. Human homologs were used for this analysis. Notably, both up- and down-regulated genes showed a statistically significant enrichment of several biological processes. Enrichment of synaptic transmission (GO: 000268; *P* = 0.002, Benjamini adjusted) and transmission of nerve impulse (GO: 000268; *P* = 0.012, Benjamini adjusted) in the down-regulated set was particularly consistent with the known AED mechanism. Next, I examined whether differentially expressed genes also over-represent genes reported in the literature as associated with AED response. The most recently published compilation of AED pharmacogenetic studies[2] was used in this analysis. Notably, two of the total eight genes reported as showing a statistically significant association, namely, *HLA-B* and *HSPA1A*, were common to the up-regulated gene set. Interestingly, both these genes represented enriched biological processes in the up-regulated set, with the total number of NaVP regulated genes in these processes being 298. Although small numbers precluded a statistical test for significance of this overlap, the trend for enrichment of pharmacogenetic candidates in differentially expressed genes is obvious considering the total number of genes in the human genome. Given the above, my analysis, in particular, supports the candidacy of *HLA-B* and *HSPA1A* in AED pharmacogenetics. In general, it supports the usefulness of transcriptomic data in pharmacogenetic association studies.
